# Molecular diversity of the *invA* gene obtained from human and egg samples

**DOI:** 10.14202/vetworld.2019.1033-1038

**Published:** 2019-07-15

**Authors:** Mona Kadry, Sara Mohamed Nader, Sohad M. Dorgham, Mai M. Kandil

**Affiliations:** 1 Department of Zoonoses, Faculty of Veterinary Medicine, Cairo University, Giza 11221, Egypt; 2 Department of Microbiology and Immunology, National Research Centre, Giza 12622, Egypt

**Keywords:** *invA* gene, phylogeny, *Salmonella*

## Abstract

**Background and Aim:**

Salmonellosis is one of the most common foodborne bacterial diseases in the world. The great majority of *Salmonella* infections in humans are foodborne with *Salmonella enterica* and *Salmonella* Typhimurium accounting for a major part of the problem. The objective of this study was to investigate the presence of *invA* gene in strains of *Salmonellae* isolated from eggs and diarrheal swabs from human cases. In addition, the relationship between *inv*A gene nucleotide sequences from different sources (human stool and egg samples) have been studied through phylogenetic tree.

**Materials and Methods:**

One hundred and seventy eggs (eggshell and its contents) and 160 stool swabs samples were collected from four poultry farms and medical hospital in Giza Governorate.

**Results:**

The study reported the presence of two *Salmonella* strains in eggshell surface with an overall isolation rate of 1.2 and 0% of the egg content. *Salmonella* Enteritidis and *Salmonella* Typhimurium were isolated from eggshell surface with an incidence of 50% for each strain. Six salmonella strains were isolated from human stool with an incidence of 3.75%; the isolated strains are *S*. Typhimurium, *S*. Enteritidis, *Salmonella* Virchow, *Salmonella* Haifa, and *Salmonella* Kentucky with an incidence of 33.3%, 16.6%, 16.6%, 16.6%, and 16.6%, respectively. Among eight *Salmonella* strains, *invA* gene was detected with percentage of 50%. The phylogenetic analysis of the sequences *invA* gene, from two isolates included in this study and five isolates retrieved from GenBank showed that sequence from human, layer hens, egg, and water in the same clusters.

**Conclusion:**

Close relation between drinking contaminated water and layer hens and contaminated water is one such source.

## Introduction

*Salmonella* is one of the most important foodborne pathogens which caused food safety hazards for the food industry. Salmonellosis is a critical medical issue and a major challenge worldwide having greater significance in developing countries [1]. The danger of *Salmonella* may vary between the production systems, caused by components of the husbandry systems affecting disease development and pathogen shedding or differences in the level of resistance to the pathogen [2].

The presence of *Salmonella* in poultry farms is a major concern for public health [3]. Poultry products, particularly eggs and egg products, are nutritive nourishment of human food. Egg farms and market outlets may be contaminated with *Salmonellae* at any production stage by horizontal or vertical transmission. Vertical transmission means contamination of egg yolk, albumin, membranes, or eggshells. While in horizontal transmission, disease is penetrated during or after oviposition through the eggshell from the gut or contaminated feces [4]. The cause and the method of the spread of *Salmonella* spp. between the egg items and the customers should be recognized to influence the illness manage. Moreover, *Salmonella* spp. contamination may be prevalent in a farm environment. The virulence of *Salmonella* is linked to a combination of chromosomal and plasmid factors. Different genes such as *invA*, *spv*, *fimA*, and *stn* are known as major virulence genes accountable for salmonellosis. The chromosomally located invasion gene *invA* codes for a protein in the inner membrane of bacteria that are necessary for invasion of epithelial cells [5]. The *invA* sequence of enteric bacteria contains sequences distinctive to the current genus and has been established as an appropriate polymerase chain reaction (PCR) target with potential diagnostic application [6,7]. Conventional methods for the identification of *Salmonella* are time-consuming and need selective enrichment and plating, followed by biochemical tests. On the other hand, PCR is a rapid and reliable technique for the detection and identification of foodborne pathogens as a complementary to conventional culture [6].

Therefore, our study was designed to investigate the presence of *invA* gene in *Salmonellae* strain. The phylogenetic tree was used to detect the relationship between the *invA* gene sequences from different sources (human stool and egg samples).

## Materials and Methods

### Ethical approval and informed consents

The study was conducted according to ethical guidelines approved by Faculty of Veterinary Medicine, Cairo University, Egypt. Stool samples from human were collected after informed consents.

### Sample collection

A total of 170 eggs (eggshell and its contents) from four poultry farms and 160 stool swabs samples from the Medical hospital were collected during the period from April to May 2016 in Giza Governorate.

### Isolation and identification of *Salmonella* strains

#### Eggshell surface

A sterile cotton swab, soaked in sterilized normal saline was swabbed on egg surface and immersed in 10 ml normal saline solution followed by transmission to 90 ml of buffered peptone water then incubated at 37°C for 18 h [8].

#### Egg albumin and yolk

Samples of egg yolks and egg albumins were examined separately as 5 ml of each sample was mixed with 5 ml of normal saline solution. Afterward, the solution was transferred to 90 ml of buffered peptone water and incubated at 37°C for 18 h. Identification of *Salmonella*: 1 ml pre-enriched sample was added in 10 ml Rappaport-Vassiliadis (RV) medium vortexed and incubated for 24±2 h at 42°C; then, loopful of each RV tube was cultured onto the surface of Xylose-Lysine Deoxycholate (XLD) agar for 24 h at 37°C.

#### The stool samples

About 2-10 g stool was collected into a sterile leak-proof container (not use preservatives) and then thoroughly coated sterile cotton swab with fecal material then inserted the swab in its sheath and these samples were inoculated aseptically into tubes containing buffer peptone water. About 1 ml pre-enriched sample was added in 10 ml RV medium vortexed and incubated for 24±2 h at 42°C; then, loopful of each RV tube was cultured onto the surface of XLD agar for 24 h at 37°C [9]. Typical *Salmonella* colonies were subjected to a series of biochemical, serological, and molecular tests for the identification of *Salmonella* spp.

#### Biochemical identification

Suspected colonies were identified using chemical tests, including Gram staining, indole, methyl red, Voges-Proskauer, citrate utilization, triple sugar iron, and lysine decarboxylation [10].

#### Serological identification

Serotyping of isolates was done in serogroup level by a standard agglutination test using O and H antisera (Difco, USA). It was performed by the central public health laboratories.

### Molecular detection of *invA* gene

#### DNA extraction

The DNA of *Salmonella* strains was extracted using the boiling method [11]. The reaction mixture consisted of 25 μl Platinum^™^ Hot Start PCR Master Mix (Invitrogen^™^), 1 μl DNA extract, 0.5 μl of each primer in the concentration of 20 pmol, and nuclease-free water up to 50 μl. The PCR for *invA* gene-specific oligonucleotide primers for *invA* gene is described in Table-1 [12]. Temperature and time conditions of the primers during PCR are shown in Table-2.

**Table 1:** Primer sequences for *invA* gene.

Target gene	Primer sequence	Size of amplified product	Reference
*InvA*	GTGAAATTATCGCCACGTTCGGGCAA TCATCGCACCGTCAAAGGAACC	284 BP	[12]

**Table 2:** PCR cycling conditions.

Initial denaturation	Denaturation	Annealing	Extension	Final extension	Number of cycles
95°C	95°C	58°C	72°C	72°C	35
10min	1min	1min	1min	10min	

The PCR products were resolved by electrophoresis on 1.5% (wt/vol) agarose gels (QIAGEN, Hombrechtikon, Switzerland).

#### Sequence analysis for invA gene

About 20 uL from each primer were aliquoted into thin-wall PCR tubes. Tubes were sent by mail to be sequenced at laboratory technology; then, the DNA sequencer was used to conduct sequencing. The whole experimental process was monitored through the Laboratory Information Management System. The results of sequencing were analyzed by BLAST web tool of the Genbank (NCBI). We performed the alignment by Multalin interface and for the construction of the phylogenetic tree by use from www.phylogeny.fr “one click mode.”

## Results

### Isolation and identification of *Salmonella* strains

From tested 170 eggs (eggshell and its contents) using biochemical and serological identification, only two *Salmonella* strains (*S*. Enteritidis and *S*. Typhimurium) were isolated from eggshell surface and also six *Salmonella* strains were isolated from 160 stool swabs samples with an incidence of 3.75%; the most prevalent serovar was *S*. Typhimurium (33.3%) followed by 16.6% of each *S*. Enteritidis, *Salmonella* Virchow, *Salmonella* Haifa, and *Salmonella* Kentucky.

### Molecular detection of *invA* gene

The PCR assay was carried out in this study for the detection of *Salmonella* invasion gene (*invA*) in eight *Salmonella* isolates and the result revealed that 50% were positive for *invA* gene in both egg (*S*. Typhimurium) and human (*S*. Virchow and *S*. Kentucky) isolates.

### Sequence analysis and phylogenetic tree

The authors illustrated in this study a robust phylogeny of *Salmonella* subspecies using the sequences of invasion (*invA*) gene, from two isolates with accession numbers (MG001904- MG001905) included in this study and five isolates retrieved from GenBank. Analysis of these sequences showed that the sequences included in this study (human and egg strain) have polytomy which placed in the same clusters with two strain retrieved from GenBank (layer hen and water strain) while the other three sequences retrieved from GenBank form a sole different cluster.

## Discussion

*Salmonella* is an important cause of foodborne (alimentary) health problems in humans [13]. Contamination of eggshell by *Salmonella* spp. can happen through fecal material, insects, and feed or even through transportation, storage, or during handling. Our study revealed that an overall isolation rate of *Salmonella* in eggshell surface and egg content were 1.2% and 0% respectively as shown in Table-3 , this may be due to fecal material contamination. Similar observations were stated by Davies and Breslin [14]. On the other side, the eggshell surface contamination increases the risk of egg contents contamination by penetration through egg surface cracks [15]. According to Table-3, *S*. Enteritidis and *S*. Typhimurium were isolated from egg samples (the most common serotype among *Salmonella* isolates), this finding supported by Edema and Atayese [16] who mentioned that hen eggs have become a principal source of *S*. Enteritidis and *S*. Typhimurium since those serotypes can colonize the ovarian tissue of hens, and in this way, it will be available inside the content of intact shell eggs.In addition to that, *Salmonella* spp. have abilit*y* to invade the cells of the follicles before ovulation and multiply themselves after 2 h of infection [12]. Mwansa *et al*. [17] reported that *S*. Typhimurium is the predominant *Salmonella* serovars isolated from human patients. There is a growing concern about human infections caused by other serovars such as *S*. Virchow which has higher abilities than other serovars to cause invasive salmonellosis; furthermore, *S*. Kentucky represents one of the non-typhoidal types of *Salmonella* species that microbiologists and public health professionals encounter from time to time. What makes *S*. Kentucky stand out and explains its public health importance is that this infectious agent has managed to develop resistance to some antibiotics. Hence, it’s tougher to treat [18]. Zou *et al*. [19] reported that *S*. Enteritidis has been most frequently involved in human salmonellosis outbreaks. According to CDC, *S*. Enteritidis was confirmed to be on the top of the laboratory – human *Salmonella* infections reports [20,21]. Although potentially all livestock can be infected with *Salmonella* species, contaminated poultry meat and eggs may be the main source of *Salmonella* infection for humans, especially where *S*. Enteritidis is implicated, human infections can be traced back to eggs and poultry meat [22]. From this result, it is suggested that there is a strong relationship between cases of *Salmonella* infection related to human illness and *Salmonella* species positive in poultry meat and so this explanation is agreed with that stated by Centers for Disease Control and Prevention [23]. More than 70% of human salmonellosis has been attributed to the consumption of contaminated chicken meat or egg. PCR has become a powerful tool for the detection of pathogens in food, especially *Salmonella* in the past decade [24]. Furthermore, the detection of *Salmonella* is rapidly and accurately due to the primer sequences that are selected from the gene *invA*. The *invA* gene considered to be the international standard for the identification of salmonellosis, in addition to being introduced as effective, rapid, and accurate method for the detection of *Salmonella* in foods of animal origin [25]. The presence of *invA* gene is referred to its ability for invasion of cells, having the capacity to invade and survive in macrophages [26]. The World Health Organization [27] mentioned that *S*. Typhimurium and *S*. Enteritidis are the most significant serovars harboring the virulence *invA* gene causing salmonellosis globally. According to our results (Table-4), among eight *Salmonella* strains, *invA* gene was detected 50%, this results fully agreed with Indu and Kashmiri [28] who mentioned that *invA* gene was present in 22 isolates (55%) from 40 *Salmonella* isolates from poultry meat samples. Our results disagreed with Sheila *et al*. [29] who stated that of 32 samples, four samples of *Salmonella* which isolated from milkfish carried *invA* gene with a length of 284 bp, while 28 samples did not show the target band. The current study explained the absence of *invA* gene in certain *Salmonella* strains because they are not invasive or may have other invasive mechanisms; this indication supported by Malorny *et al*. [25].

**Table 3:** The incidence of isolation and serological identification of *Salmonella* strains.

Source of samples	Number of examined samples	*Salmonellae* isolates	Serotyping	
	
		n(%)	Type	n(%)
Eggshell surface	170	2(1.2)	*S.* Enteritidis	1(50)
			*S.* Typhimurium	1(50)
Egg content	170	0(0)	-	-
Stool	160	6(3.75)	*S.* Typhimurium	2(33.3)
			*S.* Enteritidis	1(16.6)
			*S.* Virchow	1(16.6)
			*S.* Haifa	1(16.6)
			*S.* Kentucky	1(16.6)

*S.* Typhimurium=*Salmonella* Typhimurium, *S.* Enteritidis=*Salmonella* Enteritidis, *S.*Virchow=*Salmonella* Virchow, *Salmonella* Haifa=*S.*Haifa, *S.*Kentucky=*Salmonella* Kentucky

**Table 4:** Detection of *invA* gene from the positive serotyping.

Source of samples	Serotypes	Presence of *InvA* gene
Eggshell surface	*S.* Enteritidis	Negative
	*S.* Typhimurium	Positive
Stool	*S.* Typhimurium	(1 out of 2)
	*S.* Enteritidis	Positive
	*S.* Virchow	Negative
	*S.* Haifa	Positive
	*S.* Kentucky	Negative

*S.* Typhimurium=*Salmonella* Typhimurium, *S.* Enteritidis=*Salmonella* Enteritidis, *S.* Virchow=*Salmonella* Virchow, *Salmonella* Haifa=*S.* Haifa, *Salmonella* Kentucky=*S.* Kentucky

### Analysis of phylogenetic tree

**Figure-1:**
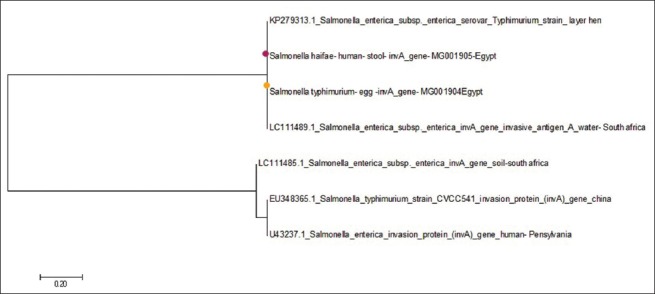
A phylogeny of *Salmonella* subspecies using the sequences of invasion *invA* gene.

Bacteria belonging to the genus *Salmonella* are a very important reason for human food poisoning. In certain parts of the world, *Salmonella* is also an important cause of disease in poultry. *Salmonella* serotypes can be introduced into poultry flocks from many different sources which can carry *Salmonella* either at the farm level or in the processing plants. We illustrated in this study a robust phylogeny (Figure-1) of *Salmonella* subspecies using the sequences of invasion (*invA*) gene, from two isolates included in this study and five isolates retrieved from GenBank. Analysis of these sequences showed that sequence from human, layer hens, egg, and water in the same clusters which refer to the close relation between drinking contaminated water and layer hens and contaminated water is one such source, which returns into the contamination of eggshell and finally leads to human infection [30]. The other cluster explains the use of fecal matter of animals and poultry in fertilizing the ground, leading to soil contamination which may also reflect on shell egg contamination on the farm [31]. According to our results, it is noted that chicken and human are an important source of infection to each other. This result was supported by Nawar and Khedr [32] who reported that *Salmonellae* can be transmitted to humans through dealing with chicken meat and/or consumption of uncooked meat and eggs.

## Conclusion

Finally, from work done here, we have to advise; application of strict biosecurity will be a need on the farm. Very strict restrictions measures on personnel and equipment movement should be taken. Routine checks for the presence of *Salmonella* species should be often undertaken. Within chicken meat processing plants, there would be a number of steps taken (e.g., water chlorination, correct temperature controls) to ensure that contamination is under control. The study concluded that eggs are representing a source of infection with *Salmonella* in the absence of exposure to sufficient heat during the process of cooking and, therefore, should beware of desserts or foods containing raw eggs. In addition to that, personal hygiene of food handlers must be taken in consideration; food areas should be kept clean and regularly sanitized to reduce the risks of food poisoning outbreaks and finally, make people aware of the dangers of foods that contain undercooked eggs and egg products.

## Authors’ Contributions

SMD and MMK performed bacterial isolation and identification. MK, SMN finished PCR and analysis of phylogenetic tree. All authors participated in draft and revision of the manuscript. All authors read and approved the final manuscript.
